# Using *Novosphingobium aromaticivorans* for Concurrent Production of Intracellular and Extracellular Products from Aromatics Extracted from Poplar Biomass

**DOI:** 10.1021/acsestengg.5c00956

**Published:** 2026-02-17

**Authors:** Bumkyu Kim, Benjamin W. Hall, Dennis V. Haak, Jason Coplien, Steven D. Karlen, Timothy J. Donohue, Daniel R. Noguera

**Affiliations:** † Department of Civil and Environmental Engineering, 5228 University of Wisconsin–Madison, 1415 Engineering Drive, Madison, Wisconsin 53706, United States; ‡ Wisconsin Energy Institute, University of Wisconsin–Madison, 1512 University Avenue, Madison, Wisconsin 53726, United States; § 282101 Great Lakes Bioenergy Research Center, 1512 University Avenue, Madison, Wisconsin 53726, United States; ∥ Department of Bacteriology, University of Wisconsin–Madison, 1550 Linden Drive, Madison, Wisconsin 53706, United States

**Keywords:** aromatic compounds, carotenoids, lignin, biomass to product, biotechnology

## Abstract

Achieving high biochemical production in biotransformations of renewable resources requires using concentrated cultures that not only generate the product of interest but also produce abundant microbial cell waste. We explored the concept of gaining value from microbial cells by producing intracellular products in tandem with a desired extracellular product. Specifically, we engineered a strain of*Novosphingobium aromaticivorans* to extracellularly produce 2-pyrone-4,6-dicarboxylic acid (PDC) from aromatic substrates and to intracellularly accumulate astaxanthin along with coenzyme Q_10_, all of which are products of industrial interest. Achieving the goal of concurrent production of intracellular and extracellular products required the creative application of bioreactor engineering principles. Although a continuously fed membrane bioreactor (MBR) maximized extracellular product biosynthesis, it had a negative effect on intracellular product accumulation. However, operating the MBR as a sequencing batch reactor (MBR-SBR) with a step-feed resulted in stable concurrent production of both extracellular and intracellular products. With aromatics extracted from poplar biomass, we achieved productivities of 1.14 g of PDC/L-h for the extracellular product and 0.04 mg of astaxanthin/L-h and 0.64 mg of CoQ_10_/L-h for intracellular products, respectively. Our findings demonstrate that the mode of operation of a bioreactor impacts the simultaneous production of intracellular and extracellular products by*N. aromaticivorans*.

## Graphical abstract

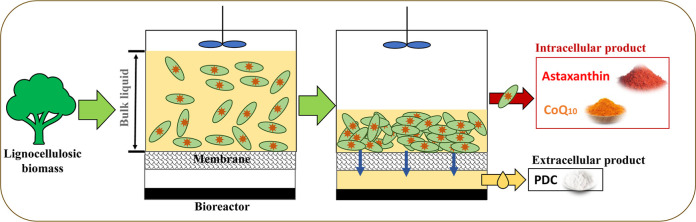

## Introduction

Society’s dependence on fossil fuels as a source of both energy and commodity chemicals is considered to be a major driver of climate change.^1^ Plant biomass, a carbon-neutral lignocellulosic renewable feedstock, and often an agricultural waste from food production, presents a promising alternative for the biosynthesis of fuels and chemicals that could potentially replace some fossil fuel use.^2–5^ In a currently envisioned scenario, cellulosic sugars could be fermented into alcohols to be used as drop in biofuels^6^ or further processed to make jet fuel,^7^ while other major components of plant biomass, such as lignin, could be transformed into biochemicals.^8–10^

In recent decades, the production of biochemicals from aromatic monomers stored in lignin has been investigated. Various effective strategies for lignin depolymerization have been developed including alkali-based, acid-based, reductive-catalytic, oxidative-catalytic, photocatalytic, thermal, and enzymatic hydrolysis treatments.^11^ One approach to biochemical production from lignin relies on generating aromatic-rich streams that can be used by microbial cultures to funnel the aromatic compounds toward the production of a single extracellular product such as 2-pyrone-4,6-dicarboxylic acid (PDC)^12,13^ or *cis*,*cis*-muconic acid (ccMA).^14,15^ These products are derived from disrupting aromatic metabolism, and thus, their production requires additional organic carbon sources (such as glucose or other organics in deconstructed biomass) to support microbial growth.

Most studies of these microbially based conversions have used either single aromatic compounds as models of lignin-derived aromatics^15–17^ or diluted mixtures of aromatics derived from biomass^10,18,19^ and have been aimed at investigating microbial transformation pathways or the effectiveness of genetic modifications to reach the desired product. Beyond these demonstrations, we are interested in process intensification and the use of productivity metrics to assess the technical and economic feasibility of the proposed processes.^20,21^ When aiming at operating processes with high production rates, there are limitations due to the solubility of aromatic compounds, the toxicity of the concentrated aromatic streams, and the need to manage high-density microbial cultures and the resulting waste products generated. In particular, achieving high production rates of extracellular products inevitably requires the operation of bioreactors capable of generating high-density cultures.^15,16^ For instance, the highest PDC titer reported to date (∼100 g/L) was possible with the use of vanillic acid as the single aromatic substrate and with a culture that reached an extremely high cell concentration that precluded continuous reactor operation.^16^ The highest PDC production rate reported to date (1.69 g/L/h)^20^ used syringic acid as the sole aromatic substrate and applied membrane separation to achieve high-density cultures. In both cases, although aromatic compounds were converted into PDC, a secondary substrate (e.g., glucose) was required to support cell growth, contributing to the accumulation of large quantities of microbial cells that in a scaled-up process would be considered waste that needs to be properly managed for its environmentally sound disposal. Embracing the reality that high-rate production of products from lignin-derived aromatics will generate high amounts of microbial cell residues, we hypothesize that it may be possible to add additional value to the microbial cells by engineering strains that accumulate valuable products intracellularly. Therefore, concurrent with the production of the extracellular product, the intracellular products can be harvested from the accumulated cells. The concept was demonstrated in earlier research^22^ with diluted aromatic streams, and here, we describe process intensification efforts that lead to sustained intracellular and extracellular product formation.

This study specifically aimed at investigating how to achieve a high productivity of both intracellular and extracellular products in a bioreactor. We engineered *Novosphingobium aromaticivorans* PDC2SastaW, a modified version of strain PDCSastaW,^22^ to produce PDC extracellularly and two valuable intracellular products, astaxanthin and coenzyme Q_10_ (CoQ_10_). We utilized a membrane bioreactor (MBR) to enhance the productivity of PDC, increase biomass retention, and produce a cell-free effluent.^20^ The parent strain, *N. aromaticivorans* DSM12444 (also referred to as strain F199), is a genetically tractable alphaproteobacterium originally isolated from deep subsurface sediments.^23,24^ It is known to degrade a diverse set aromatic compounds^23^ including most types of aromatic compounds derived from lignin^12,25^ and to break down interunit linkages in lignin.^26,27^ With the *N. aromaticivorans* PDC2SastaW strain, we demonstrate that a sequencing batch reactor (SBR) with step-feed operation and integrated to a MBR system creates conditions that improve the intracellular accumulation of astaxanthin and CoQ_10_ while maintaining high production of the extracellular product PDC.

## Results and Discussion

A previously described MBR system^20^ was used in these experiments. All bioreactor runs were initiated in batch mode, with *N. aromaticivorans* PDC2SastaW as the inoculum and 50 mM (9 g/L) glucose as the sole carbon source in the standard mineral base (SMB) medium.^20^ This initial enrichment step was followed up by a flow-through operation with growth medium and operational conditions adjusted as described below for each experiment. A summary of all experiments can be found in Table S1.

### Production of Intracellular Products in a Continuous Flow MBR System

We first evaluated whether the operational conditions that were found to be optimal for extracellular PDC production in an earlier study^20^ were applicable for stable production of intracellular products (Figure 1). For this, we evaluated a continuous flow operation with 50 mM glucose as the sole carbon source and assessed the accumulation of astaxanthin as a test intracellular product. The flow rate was initially set to 8.33 mL/h, corresponding to a hydraulic retention time (HRT) of 24 h in a 200 mL bioreactor. Subsequently, the HRT was decreased to 12 h and then to 6 h (Figure 1). As expected from the use of membranes to separate and retain the cells in the bioreactor,^20^ the optical density of the culture (OD_600_) increased during bioreactor operation. However, the concentration of astaxanthin per unit of dry cell weight (dcw) gradually decreased as the density of the microbial culture increased (Figure 1A). For comparison, we measured the concentration of CoQ_10_, another intracellularly stored product of industrial interest, which also decreased during this bioreactor run (Figure 1B). The relative amount of astaxanthin decreased from 0.07 mg/g_dcw_ at the beginning of the experiment to 0.01 mg/g_dcw_ at the end, and the relative amount of CoQ_10_ decreased from 0.46 mg/g_dcw_ to 0.05 mg/g_dcw_.

**Figure 1:**
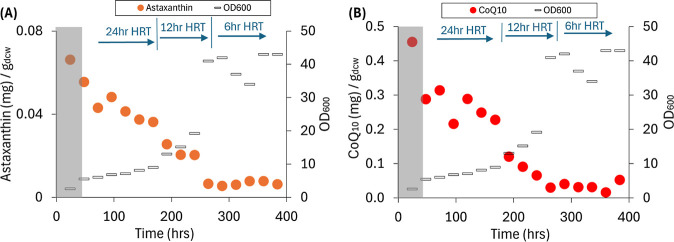
Intracellular product accumulation in experiments using 50 mM glucose and no aromatic substrate. The bioreactor was operated as a continuous flow MBR system and using NH_4_OH for pH control. (A) Relative amount of astaxanthin and the cell density throughout the operational period. (B) Relative amount of CoQ_10_ and the cell density throughout the operational period. The gray-colored boxes indicate the initial batch mode period of bioreactor operation. The HRT transitions occurred at 168 and 240 h, respectively.

One possibility for the decreasing accumulation of astaxanthin is degradation of this carotenoid upon prolonged incubation. To test this, experiments in which we incubated a high-density culture with continuous aeration and without the addition of a carbon source for ∼300 h showed only a negligible reduction in the relative accumulation of astaxanthin (Figure S1). From this, we conclude that astaxanthin degradation does not occur during endogenous respiration.

Furthermore, since the high cell concentrations in the continuous-fed system create a low glucose-to-cell ratio, we tested the hypothesis that a low glucose concentration might negatively impact astaxanthin production. However, batch experiments with different glucose-to-cell ratios showed that a low glucose-to-cell ratio led to increased astaxanthin production (Figure S2), proving the hypothesis to be false. The results of increasing astaxanthin production at low glucose-to-cell ratios are in agreement with studies with a strain of the yeast *Phaffia rhodozyma*, which has the same carotenoid biosynthesis pathway as *N. aromaticivorans*,^28^ in which a low glucose-to-cell ratio increased the relative amount of astaxanthin in a cell.^29^ The accumulation of carotenoids is widely recognized as a microbial mechanism of stress response, primarily functioning against oxidative stress.^30^ A low glucose-to-cell ratio limits substrate availability per cell, which may suppress cellular metabolism and reduce oxygen consumption. This reduction leaves cells exposed to higher dissolved oxygen levels, likely increasing the level of oxidative stress. Consequently, it is plausible that *N. aromaticivorans* enhances astaxanthin accumulation under low glucose-to-cell ratio conditions as an oxidative stress response, which could explain our observations.

Thus, we conclude from these experiments that continuous flow operation of bioreactors is not optimal for concurrent production of intracellular and extracellular products and that improvement in intracellular product accumulation requires a different mode of bioreactor operation.

### Production of Intracellular Products in a Sequencing Batch Reactor-MBR System

Since the intracellular products were highest at the beginning of the experiments, when the reactor runs were initiated in batch mode (Figure 1), we hypothesized that intracellular product accumulation would be higher if the bioreactor was operated as a sequencing batch reactor (SBR). The operation of a conventional SBR includes a series of sequential steps: fill, react, settle, and draw.^31^ Combining MBR and SBR, we defined a bioreactor cycle consisting of 4 stages: (1) feeding by rapid addition of fresh medium to reach a 250 mL working volume; (2) batch growth stage ending when the organic substrate was depleted, as signaled by the sudden increase in dissolved oxygen concentration; (3) membrane filtration to remove the medium and concentrate the cell material; and (4) harvest of approximately 95% of the concentrated cells to recover the intracellular product (Figure S3). This cycle can be repeated for continuous operation of the SBR-MBR system.

In an initial experiment with these sequencing batch conditions and 50 mM glucose as the only organic substrate, five cycles were conducted to evaluate the production of intracellular products (Figure 2A). We found that the accumulation of intracellular products was reproducible from cycle to cycle (Figure 2A). At the beginning of the cycle, each batch started with an initial OD_600_ of 2.6 ± 0.2 and reached a final OD_600_ of 11.5 ± 0.5 at the end of the growth stage (Figure 2B). The removal of the spent medium through the filtration process produced 160 ± 17 mL of filtered effluent and 90 ± 4 mL of concentrated cells, which reached an OD_600_ of 26.5 ± 2.2, about 10-times higher than the initial OD_600_ (Figure 2B). After cell harvesting, the extraction of intracellular products produced 0.04 ± 0.01 mg of astaxanthin/g_dcw_ and 0.44 ± 0.04 mg of CoQ_10_/g_dcw_ (Figure 2A), which are comparable to the values of intracellular product accumulation during the initial batch growth in the previous experiment (Figure 1).

**Figure 2:**
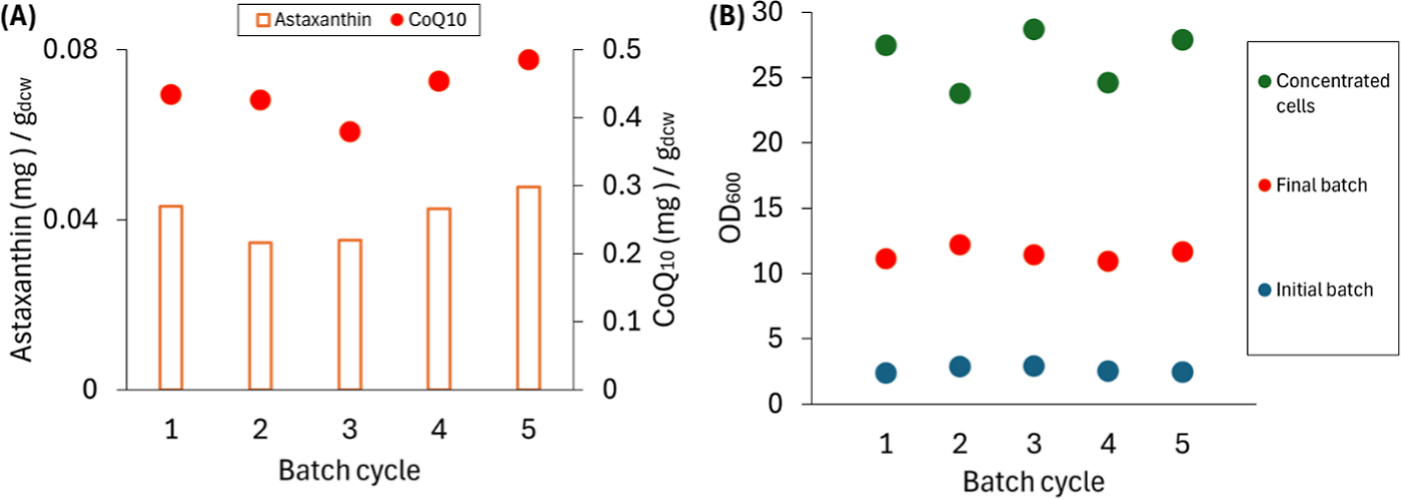
Intracellular product accumulation and cell density during bioreactor operation as an SBR-MBR system, with media containing 50 mM glucose and no aromatic substrate, and using NH_4_OH for pH control. (A) Astaxanthin and CoQ_10_ accumulation at the end of each batch cycle. (B) Cell density at the starting and end of react period of each cycle, and after concentrating the cells by filtration.

This SBR-MBR experiment showed the feasibility of the stable production of intracellular products throughout multiple operational cycles. Based on these results, we proceeded to evaluate the SBR-MBR system for concurrent production of extracellular (PDC) and intracellular (astaxanthin, CoQ_10_) products.

### Concurrent Production of Intracellular and Extracellular Products in the SBR-MBR System

To evaluate the concurrent production of intracellular and extracellular products, we used a synthetic media containing *p*-hydroxybenzoic acid (*p*HBA) as an aromatic compound that can be extracted from poplar biomass and channeled to PDC production with engineered strains of *N. aromaticivorans*.^10,20^ The startup period in this reactor included a batch enrichment step with glucose as the sole organic source, followed by an initial 1 h flow-through acclimation step in which the medium was supplemented with approximately 3 mM (0.41 g/L) *p*HBA to ensure increased expression of genes related to aromatic metabolism. Then, the feed and operational conditions were adjusted as described below.

To test for PDC concentrations that the SBR-MBR system could achieve, we performed an experiment consisting of eight cycles, with increasing *p*HBA concentrations in each subsequent cycle (i.e., 0, 2, 10, 15, 23, 25, 30, and 35 mM) and with 50 mM glucose. We used NH_4_OH as the base to neutralize the acidic aromatic substrate (*p*HBA) and maintain the pH during production of the dicarboxylic acid (PDC). Previous bioreactor experiments with *N. aromaticivorans* strains engineered to produce PDC from *p*HBA have shown that extracellular accumulation of the intermediate protocatechuic acid (PCA) signals cellular stress.^20^ Therefore, we monitored the concentrations of NH_4_^+^, *p*HBA, PCA, PDC, and glucose at the end of each cycle (Figure 3).

**Figure 3:**
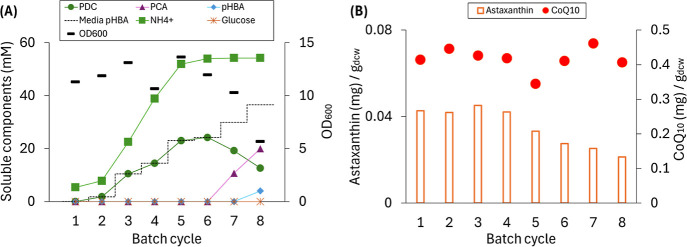
Performance data for a batch flow-through MBR fed increasing concentrations of *p*HBA in each batch cycle (0 to 35 mM *p*HBA) and 50 mM glucose, using NH_4_OH for pH control. (A) Concentrations of *p*HBA, PCA, PDC, glucose, and cell density at the end of each batch cycle. (B) Relative amount of astaxanthin and CoQ_10_ at the end of each batch cycle.

Regarding extracellular production (Figure 3A), an ∼100% molar PDC yield from *p*HBA was achieved in cycles 2 to 6, resulting in a stable production of up to 24.2 mM PDC when 25 mM *p*HBA was used (Figure 3A). In cycles 7 and 8, which received 30 and 35 mM *p*HBA, respectively, incomplete aromatic conversion to PDC was observed, with accumulation of the intermediate PCA in both cycles, and residual *p*HBA remaining at the end of cycle 8 (Figure 3A). In the case of intracellular products (Figure 3B), the accumulation of CoQ_10_ during the eight cycles was stable, whereas astaxanthin accumulation decreased when using higher *p*HBA concentrations, starting at cycle 5 (Figure 3B). The average accumulation of CoQ_10_ in the 8 cycles was 0.42 ± 0.03 mg of CoQ_10_/g_dcw_. The average accumulation of astaxanthin in the first 4 cycles was 0.04 ± 0.00 mg of astaxanthin/g_dcw_. About a 50% reduction in the relative amount of astaxanthin was observed in cycles 7 and 8, compared to cycles 1 to 4 (Figure 3B). Furthermore, although the residual glucose concentration in the latter cycles remained close to zero, the cell concentrations decreased with increasing *p*HBA concentrations (OD_600_ = 10.3 in cycle 7 and 5.7 in cycle 8). The NH_4_^+^ concentration gradually increased along with the increase of the feed *p*HBA concentration up to cycle 6 and did not increase in cycles 7 and 8 (Figure 3A). Taken together, these results are indicative of cellular stress impacting accumulation of both intracellular and extracellular product under the higher pHBA conditions used in cycles 7 and 8.

We have observed that PDC production with *N. aromaticivorans* is affected by the accumulation of counterions when using a base for pH control in bioreactor operation, leading to accumulation of protocatechuic acid (PCA).^20^ In prior work, when using NH_4_OH for pH control, PCA accumulated when NH_4_^+^ concentrations exceeded 100 mM.^20^ In this experiment, the NH_4_^+^ concentration in cycles 6, 7, and 8 remained ∼54 mM, which is below the previously observed inhibitory levels. This suggests that the accumulation of PCA in the last cycles was not caused by NH_4_^+^ accumulation.

Another intriguing observation in these experiments was that CoQ_10_ production remained stable, whereas astaxanthin production decreased (Figure 3B). One hypothesis for this observation is that the carbon-to-nitrogen (C/N) ratio may affect astaxanthin production during growth. Experiments with astaxanthin-producing microalgae *Dunaliella viridis* and astaxanthin-producing yeast *Rhodotorula glutinis* have shown a negative relationship between nitrogen concentration in the media and carotenoid production.^32,33^ The experiments with *N. aromaticivorans* PDC2SastaW showed that during bioreactor operation, the media nitrogen concentrations gradually increased (Figure 3A). This resulted in a reduction of the C/N ratio during multiple cycles of reactor operation. For example, the initial C/N ratios of cycles 4, 5, 6, 7, and 8 in Figure 3 gradually decreased as follows: 0.15, 0.12, 0.11, 0.09, and 0.07. Therefore, the reduction in astaxanthin production might be related to the decreasing C/N ratio in batch growth. This could potentially be alleviated by replacing NH_4_OH with another base (NaOH for example) for pH control. However, this strategy may not be fruitful since the inhibitory effect of Na^+^ on the production of extracellular PDC has been shown to occur at lower concentrations than the apparent inhibition by high concentrations of NH_4_^+^.^20^ For instance, a 20% reduction in growth rate was previously observed in the presence of 60 mM NaCl, in comparison to 60 mM NH_4_Cl.^20^

Another possible explanation for why CoQ_10_ production was not affected by the low C/N ratio experienced during SBR-MBR operation may be related to the biosynthesis of CoQ_10_, which involves the production of a quinone head and an isoprene tail.^34–36^ The biosynthesis of the quinone head uses *p*HBA as an early pathway intermediate,^37^ which suggests that exogenously supplied *p*HBA may influence CoQ_10_ synthesis in *N. aromaticivorans*. This is supported by studies with *Sporidiobolus johnsonii*, which showed that the exogenous addition of *p*HBA can increase CoQ_10_ production almost 8 fold.^38^ Therefore, the stable CoQ_10_ production in this study may have resulted from the use of a small fraction of exogenous *p*HBA for the synthesis of this compound.

A final hypothesis to explain the results of the SBR-MBR operation is that PCA accumulation in these experiments may be due to the sudden exposure of the culture to high *p*HBA concentrations during the feeding stage of the SBR cycle. We experimentally evaluated this hypothesis by modifying the feeding and growth stages of the cycle, as described below.

### Concurrent Production of Intracellular and Extracellular Products in an SBR-MBR System with Step-Feed Operation

The objective of this experiment was to reduce the sudden addition of *p*HBA at the beginning of a cycle while simultaneously increasing the *p*HBA concentration in the feed. This was achieved by converting the feeding and growth stages of the SBR cycle to a fed-batch stage. That is, rather than rapidly adding fresh medium to reach the 250 mL working volume, smaller volumes of the medium were added intermittently in order to ensure that *p*HBA media concentrations remained below ∼20 mM. Through this approach, intermittent additions of medium were made until the 250 mL working volume was reached (Figure 4).

**Figure 4:**
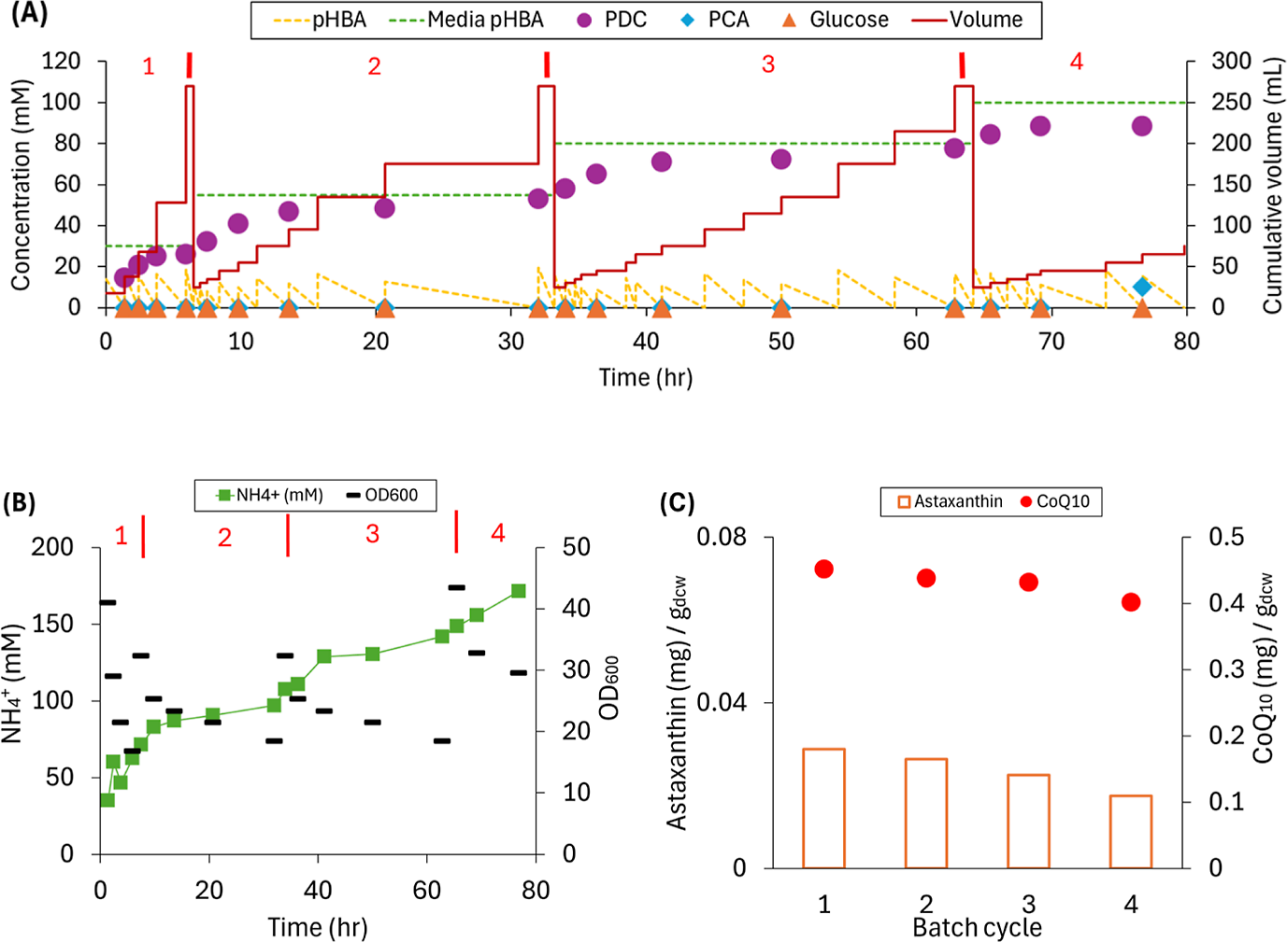
Performance data for a step-fed SBR-MBR operation with increasing *p*HBA concentrations (35, 50, 80, and 100 mM) and increasing glucose concentrations (45, 65, 90, and 110 mM) at each cycle and using NH_4_OH for pH control. The starting times for each cycle were 1.4, 6.5, 33.2, and 64.2 h, respectively. (A) Concentrations of *p*HBA in the media, estimated concentration of *p*HBA in each feeding step, and measured PCA, PDC, and glucose concentrations at different times during the cycles. The cumulative volume throughout the operational period of four batch cycles is also shown. (B) Concentration of NH_4_^+^ and the cell density during the four batch cycles. (C) Relative amounts of astaxanthin and CoQ_10_ at the end of each batch cycle.

This experiment consisted of four cycles with increasing *p*HBA concentrations at each cycle, starting from 30 mM, which is near the maximum tested in the prior experiment (Figure 3), and aiming to reach 100 mM, the maximum tested in an earlier MBR operation^20^ (Figure 4A). The glucose concentrations in each cycle were also increased (45 mM, 65 mM, 90 mM, and 110 mM, respectively) to support cell carbon and energy needs. This strategy was effective at preventing PCA accumulation during the growth stage and allowing the use of higher feed *p*HBA concentrations (Figure 4). PDC accumulation in each cycle was observed in a nearly stoichiometric fashion with produced PDC concentrations approximating feed *p*HBA concentrations at the end of each cycle during the first three cycles. By the end of the cycle 4, 88.6 mM PDC was obtained from 100 mM pHBA in the feed solution with the remaining aromatic accumulated as PCA (Figure 4A).

The accumulation of CoQ_10_ throughout the duration of the experiment averaged 0.43 ± 0.01 mg/g_dcw_, and astaxanthin accumulation averaged 0.02 ± 0.01 mg/g_dcw_ (Figure 4C), which are close to their highest values in the previous batch flow-through MBR experiment (Figure 3). Thus, compared with the rapid addition of fresh medium in SBR operation (Figure 3), the step feeding strategy did not decrease intracellular product accumulation while allowing for an ∼4× increase in accumulation of the extracellular product.

### Concurrent Production of Intracellular and Extracellular Products with Aromatics Derived from Poplar Biomass

Based on these results, we evaluated the step feeding strategy using aromatic streams from poplar biomass obtained by using alkaline pretreatment liquors concentrated by extraction (eAPL) (Figure 5). In prior work, we found that a continuous flow reactor could maintain stoichiometric production of PDC from poplar eAPL that contained ∼50 mM *p*HBA as the main aromatic substrate.^20^ Thus, the goal of these experiments was to test if a step-feeding cycle with *N. aromaticivorans* PDC2SastaW and poplar eAPL could be used to concurrently produce ∼50 mM PDC and the same intracellular product levels obtained when using *p*HBA-based synthetic media that sustained ∼50 mM PDC production (i.e., cycle 2 in Figure 4).

**Figure 5:**
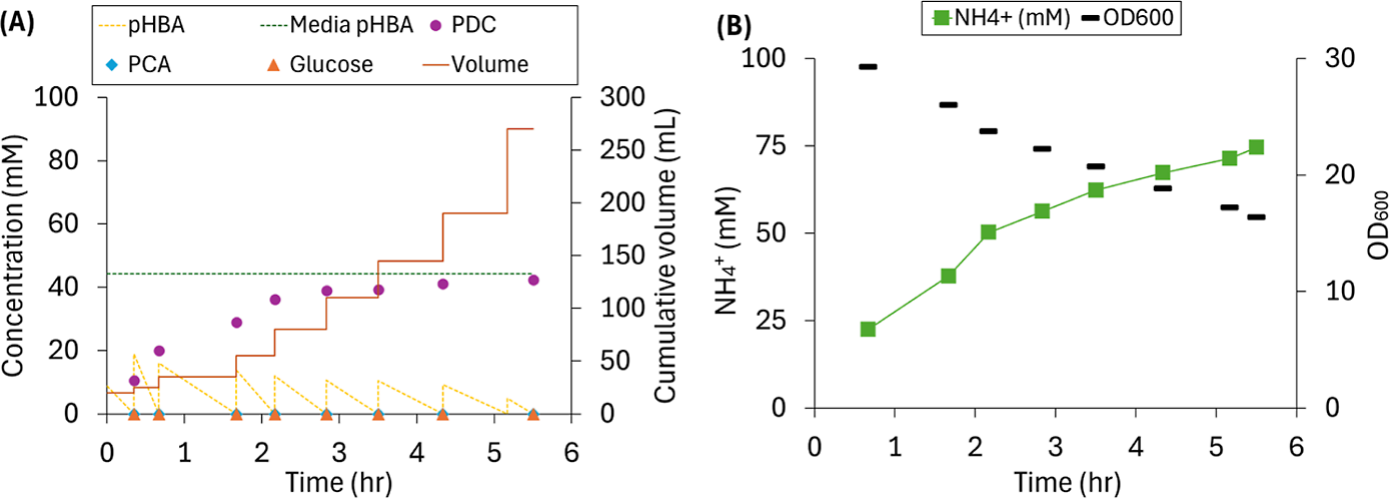
Performance data through one cycle of step-fed SBR-MBR operation using the modified poplar eAPL2 containing *p*HBA as the main aromatic compound (44.3 mM), glucose as the nonaromatic compound (50 mM), and using NH_4_OH for pH control. (A) Estimated concentration of *p*HBA at the beginning of each feeding step, *p*HBA in the media, and measured PCA, PDC, and glucose concentrations. The cumulative volume throughout the fed-batch period is also shown. (B) Concentration of NH_4_^+^ and the cell density throughout the fed-batch period.

An initial test was performed with poplar eAPL that contained 83 mM *p*HBA as the main aromatic substrate, 343 mM NH_4_^+^, and 100 mM glucose (Table 1). This *p*HBA concentration was the highest we achieved when aiming for a 100 mM concentration, the maximum concentration tested in the prior experiment (Figure 4). With this eAPL, the step-feeding approach led to the accumulation of only 27 mM PDC and incomplete utilization of *p*HBA, indicating inhibition of aromatic metabolism (Figure S4). When the experiment was stopped, the concentration of NH_4_^+^ was as high as 230 mM, a level expected to inhibit the growth of *N. aromaticivorans*.^20^ In the preparation of this eAPL, NH_4_OH was used to neutralize *p*HBA and other acids present in the media. The resulting ratio of NH_4_^+^ to *p*HBA (343 mM NH_4_^+^ to 83 mM *p*HBA) was approximately 4.1:1, much higher than the 1:1 ratio observed in the synthetic medium and anticipated from neutralization of monocarboxylic acid *p*HBA. This indicated the presence of unidentified acids or buffers that accumulated during the eAPL preparation from the poplar biomass as potential inhibitors of microbial conversion.

**Table 1:** Characteristics of Alkaline Pretreatment Liquors (APL) Produced from Poplar Biomass

APL	*p*HBA (mM)	glucose (mM)	NH_4_^+^ (mM)	NH_4_^+^/*p*HBA
eAPL	83	100	343	4.1
eAPL2	44	50	50	1.1

Accordingly, modifications to the poplar eAPL preparation were made to decrease the concentration of potential inhibitors. This modified eAPL media (eAPL2; Table 1) contained 44.3 mM *p*HBA, 50 mM NH_4_^+^, and 50 mM glucose, corresponding to an NH_4_^+^ to *p*HBA ratio of 1.1:1, like the ratio in the synthetic medium and having a *p*HBA concentration ∼50 mM *p*HBA, the maximum that has been successfully used when testing extracellular production only.^20^ With this poplar eAPL2, it was possible to reach a final PDC concentration of 42.4 mM from 44.3 mM pHBA (Figure 5A). In addition, the final NH_4_^+^ concentration rose to only 74.7 mM after use of NH_4_OH to maintain pH during reactor operation (Figure 5B), below the concentration shown previously to be inhibitory.^20^ At the end of this fed-batch cycle, the relative amounts of astaxanthin and CoQ_10_ were 0.03 ± 0.01 mg/g_dcw_ and 0.44 ± 0.03 mg/g_dwc_, respectively, comparable to the values from experiments with the synthetic *p*HBA medium (Figure 4), thus achieving the goal of the experiment.

### Implications for Lignocellulosic Biorefining

A successful lignocellulosic biorefining industry would benefit from the simultaneous production of multiple high-value biochemicals and biofuels.^39,40^ In a lignocellulosic biorefinery, it is often expected that most of the carbohydrates stored in the cellulose and hemicellulose polymers will be used for biofuel production, whereas noncarbohydrate components, such as lignin aromatics, would be used for production of biochemicals.^41^ Under this premise, significant research has concentrated on the production of biochemicals from the aromatic compounds that can be recovered from lignin and other plant biomass components.^42^ Chemical treatments of either plant biomass or extracted lignin enable the release of low molecular weight aromatics that can either be separated and recovered if they have intrinsic value or microbially transformed to a desired final product. The latter is particularly useful if the chemical treatment results in a large array of water-soluble aromatics that are difficult to separate or do not have direct industrial application but can be funneled using engineered microbial catalysts toward a single product.^12,13,43–47^

Some biochemicals that have been targeted for microbial production from plant-derived aromatics include PDC,^12,13,47^ *cis*,*cis*-muconic acid,^14,48^ and adipic acid,^49^ all extracellular compounds that are secreted after engineering aromatic metabolism. In addition, intracellular accumulation of lipids and fatty acids has also been proposed.^50,51^ Several microbial chassis have been studied for these transformations, including *N. aromaticivorans*, *Pseudomonas putida*, *Rhodococcus opacus*, *Rhodospirillum toruloides*, and *Sphingobium lignivorans*, among others.^21^

As microbial strains are engineered to produce specific extracellular products from aromatic substrates, their industrial application will eventually depend on achieving high productivity with aromatic-rich streams obtained from bioenergy and other crops. Therefore, previous studies have focused on increasing productivity, where the use of high-density cultures is inevitable for enhancing production rates.^15,16,20^ Hall et al. recently proposed taking advantage of this by engineering strains to simultaneously accumulate extracellular and intracellular products.^22^ They focused on carotenoids as valuable intracellular products that are easily recovered from microbial cells^52^ and demonstrated that *N. aromaticivorans* could be engineered to accumulate different carotenoids such as zeaxanthin, astaxanthin, and β-carotene.^22^ In addition, they noted that the valuable product CoQ_10_ is also extracted from microbial cells during extraction of carotenoids. With a strain engineered to produce PDC and astaxanthin (*N. aromaticivorans* PDCSastaW^22^) and using APL obtained from sorghum, they demonstrated simultaneous production of 2.9 mM PDC (0.53 g/L), 0.11 mg of astaxanthin/g_dcw_, and 0.15 mg of CoQ_10_/g_dcw_ (Table 2). In this study, we used an altered version of an astaxanthin-accumulating and PDC-producing strain (PDC2SastaW) to investigate whether we could increase the productivity of extracellular and intracellular products by using bioreactor systems and operational conditions designed to increase cell concentration and utilize plant-derived aromatic streams with higher concentrations of aromatic and nonaromatic substrates.^20^ Using APLs concentrated by extraction and prepared from poplar biomass, we achieved 42.4 mM PDC (7.8 g/L), 0.03 mg of astaxanthin/g_dcw_, and 0.44 mg of CoQ_10_/g_dwc_. Regarding production rates (Table 2), in the batch experiments of Hall et al. with sorghum APL,^22^ they achieved 0.01 g of PDC/L-h, 0.001 mg of astaxanthin/L-h, and 0.001 mg of CoQ_10_/L-h. In this study, from poplar eAPL2, we achieved 1.14 g of PDC/L-h for the extracellular product, 0.043 mg of astaxanthin/L-h, and 0.64 mg of CoQ_10_/L-h (Table 2). Thus, optimizing bioreactor performance and using concentrated APL, the PDC titer increased ∼15-fold, the astaxanthin yield (mg astaxanthin/g_dcw_) decreased ∼4 fold, and the CoQ_10_ yield (mg CoQ_10_/g_dcw_) increased ∼3 fold. However, although the astaxanthin yield decreased compared to the study of Hall et al., the astaxanthin production rate increased ∼43 fold because of the high-density cultures that were created with the bioreactor conditions used. Furthermore, the higher CoQ_10_ yield in this study compared to that of Hall et al.^22^ (Table 2) could be due to their use of sorghum APL, in which *p*-coumaric and ferulic acids, instead of *p*HBA, were the main aromatic substrates, and thus, exogenous supply of *p*HBA for CoQ10 synthesis^37^ did not occur. Increasing the astaxanthin yield in *N. aromaticivorans* strains may require genetic engineering approaches to increase the flux of carbon through the carotenoid producing pathways. This could be achieved by overexpression of rate-limiting enzymes in the pathways for precursor formation, introduction of heterologous pathways, or placing the native pathways under the control of alternative promoters.^53^ These strategies and others have been demonstrated in tractable *Escherichia coli* strains.^54–56^

**Table 2:** Summary of PDC, Astaxanthin, and CoQ_10_ Production

			PDC titer		yield (mg/g_dcw_)		production rate				
experiment	reaction time (h)	main aromatic substrate (mM)	mM	g/L	astaxanthin	CoQ_10_	PDC (g/L-h)	Astaxanthin (mg/L-h)	CoQ_10_ (mg/L-h)	OD_600_	reference
poplar eAPL2 (Figure 5)	5.5	*p*HBA (44.3)	42.4	7.8	0.03	0.44	1.14	0.043	0.64	16	this study
*p*HBA-based synthetic media (Figure 4, cycle 2)	25	*p*HBA (50)	50.0	9.2	0.03	0.43	0.32	0.010	0.16	18	this study
sorghum APL	50	coumaric and ferulic acids (2.5)	2.9	0.5	0.11	0.15	0.01	0.001	0.001	4	Hall et al.[Bibr ref22] 2023
poplar eAPL	6	*p*HBA (50.0)	50.0	9.2	-	-	1.53	0	0	15	Kim et al.[Bibr ref20] 2024

Ultimately, the success of lignocellulosic biorefining for the production of biochemicals from plant-derived aromatic compounds at high rates and with high titers will depend on processes that are upstream of microbial upgrading and produce the aromatic streams. These processes need to produce streams that are compatible with the microbial growth. We show in this study that some of that compatibility may be achieved by adequately selecting the mode of bioreactor operation, but most of the compatibility will depend on how concentrated the aromatic streams are and what type of inhibitory substrates are present in the aromatic solutions that will be fed to the bioreactors. These issues have been extensively studied in relation to the fermentation of carbohydrate hydrolysates used for alcohol production^57,58^ but not sufficiently studied when the goal is to produce biochemicals from aromatic streams. Few studies have evaluated biochemical production from highly concentrated streams of plant-derived aromatics,^21^ and studies showing the highest titers of aromatic-derived chemicals have been performed with synthetic media using of-the-shelf aromatics.^16^ In this study, efforts were made to identify the conditions required to produce high product concentrations. The transition to step-fed operation allowed increases in PDC titers up to 88.6 mM (16.3 g/L) when using synthetic media with 100 mM *p*HBA (Figure 4A). However, when using the first version of poplar eAPL that we produced (Figure S4), the PDC titer was as low as 23 mM (4.2 g/L) even though the *p*HBA concentration in this eAPL was 83 mM. When the eAPL production methodology was modified, the PDC titer could be increased to 42.4 mM (7.8 g/L) with an eAPL (eAPL2) that had 44.3 mM *p*HBA (Table 2). Thus, more work is needed to successfully integrate the upstream production of plant-derived aromatics with a microbial upgrading step.

The compatibility of biomass-derived aromatic streams with microbial upgrading also depends on the microbial chassis used for the biological step.^21^ Regarding *N. aromaticivorans* specifically, this and previous studies^20,21^ have shown that the concentration of cations in the aromatic streams affects the activity of *N. aromaticivorans*. High cation concentration in the preparation of APLs results from the need have alkaline conditions to release aromatics that are bound to plant polymers via ester bonds and from the need to neutralize the APLs before microbial transformations take place,^20,21^ and thus, further strain optimization by genetic engineering to increase resistance to osmotic pressure^59–61^ or adaptive laboratory evolution^62^ is necessary to increase *N. aromaticivorans*’ resistance to high concentrations of cations and possibly other inhibitory substance that may increase in concentration as more concentrated aromatic streams are produced.

Finally, obtaining high product yields from the aromatics present in the plant-derived streams is also imperative to taking advantage of the effectiveness of upstream biomass deconstruction and aromatic extraction procedures. The type of upstream process and biomass feedstock dictate the characteristics of the extracted aromatics. The research presented here focused on poplar biomass and on *p*HBA as the main aromatic in the eAPL produced from poplar, but we expect that the microbial transformations described here would also apply when using biomass from other energy crops such as switchgrass or sorghum. APLs produced from grasses are expected to have high concentration of ferulic acid and *p*-coumaric acid instead of *p*HBA,^19,63^ two aromatic compounds that are metabolized by *N. aromaticivorans* ^25^ and that can also be converted to PDC by engineered *N. aromaticivorans* strains.^19,63^ In addition, the range of aromatic products that *N. aromaticivorans* can metabolize is an area of active research and for which progress is continuously made.^62,64–66^ These advances in scientific knowledge will ultimately need to be combined with engineering strategies for effective bioreactor operation to achieve the goal of having sustainable processes to produce aromatic-derived biochemicals from biomass, which is the largest renewable source of aromatic compounds on the planet.

The proposed step-feed SBR-MBR incorporates distinct operational modifications compared to a traditional SBR system, specifically utilizing a step-feed strategy during the fill phase and membrane filtration for the decant phase. Although the proposed SBR-MBR bioreactor system for simultaneous intracellular and extracellular production is more complex than the simpler MBR system used for producing only extracellular product,^20^ there is precedent and motivation from advanced bioreactor systems currently in use in the water treatment industry, where online sensors are used for control of bioreactor conditions.^67,68^ SBR is an established technology,^69,70^ and scaled-up membrane modules for cell and water separation have been extensively used.^71,72^

The technoeconomic potential of producing PDC from lignin-derived aromatics using *N. aromaticivorans* has been previously evaluated.^10,73,74^ These analyses have shown a progressive reduction in the estimated minimum selling price (MSP) of PDC as different improvements in unit processes within the biomass-to-PDC process pipeline are made. The initial analysis identified biomass deconstruction and lignin depolymerization as the critical steps that needed improvements.^10^ Further developments in these processes reduced the estimated MSP by 29%,^73^ and a further 24% reduction was achieved by reducing the amount of solvent used in biomass fractionation, with the most recent MSP estimate being at $13.98 per kg of purified PDC.^74^ The study described here uses a different aromatic extraction procedure and adds the potential production of intracellular products in addition to extracellular PDC. These are strong motivations for future technoeconomic analyses to determine the impact of these innovations.

## Conclusions

This study explored the impact of bioreactor operational modes on conditions that allowed for the stable production of an extracellular product (PDC) and two intracellular products (astaxanthin and CoQ_10_). A combination of SBR, MBR, and step feeding mode proved beneficial for the stable production of the three products. Emphasis was placed on testing operation when feeding aromatic streams with high aromatic concentrations to explore the limits to extracellular product titers. When comparing productivities with synthetic media prepared with *p*HBA as the aromatic substrate and eAPL2 produced from poplar, that also had *p*HBA as the main aromatic compound, our experiments showed higher production rates with the eAPL2 media when both media contained ∼50 mM *p*HBA, primarily because reaction times with eAPL2 were much faster than with synthetic media. However, we found that the method of preparation of eAPL strongly affects process performance because of inhibitory substances that may be present in the eAPL, or the amount of base required to balance the pH before microbial incubations. Maintaining a low NH_4_^+^ appeared to benefit both PDC and astaxanthin production, whereas production rates of CoQ_10_ did not appear to be affected by NH_4_^+^ concentration. Thus, this study demonstrated that it was possible to establish bioreactor operational conditions for stable production of extracellular and intracellular products, potentially valorizing the large amounts of cell biomass generated during biotransformation. Future technoeconomic analyses and life cycle assessments will be important to establish whether the concurrent production of intracellular and extracellular products provides economic and environmental advantages to the production of extracellular products only.

## Experimental Methods

### Bacterial Strain and Culture Inoculation

An engineered astaxanthin-accumulating *N. aromaticivorans* was utilized in this study. This strain, PDC2SastaW, was constructed from the parent strain *N. aromaticivorans* PDCSastaW, which has been previously described.^22^ Compared to the parent strain, PDC2SastaW has an additional deletion of Saro2861 (*ligM*) created by homologous recombination using a variant of the pK18*mobsacB* plasmid^22^ (detailed methods in the Supporting Information).

Frozen (−80 °C) stocks of *N. aromaticivorans* PDC2SastaW were used for overnight incubation under the following conditions: 30 °C, 200 rpm, and 10 mL of standard mineral base (SMB) medium with 20 mM (3.6 g/L) glucose. Approximately 4 mL of the overnight culture was inoculated to reactor experiments.

### Standard Mineral Base Medium

The SMB medium^26^ was prepared by adding the following three stock solutions to 1L of distilled water: 40 mL of phosphate buffer stock and 20 mL of trace mineral stock. The phosphate buffer stock included 0.5 M Na_2_HPO_4_ and KH_2_PO_4_. One liter of trace mineral stock, also called Hunter’s vitamin-free concentrated base, included 14.45 mg of MgSO_4_, 3.34 mg of CaCl_2_·2H_2_O, 9.25 mg of (NH_4_)_6_Mo_7_O_24_·4H_2_O, 99 mg of FeSO_4_·4H_2_O, 55 mg/L nicotinic acid, 24 mg of thiamin·HCl, 0.5 mg of biotin, and 50 mL of Metals “44” solution. One hundred milliliters of Metals “44” included 250 mg of ethylenediaminetetraacetic acid, 1095 mg of ZnSO_4_·7H_2_O, 500 mg of FeSO_4_·7H_2_O, 154 mg of MnSO_4_·H_2_O, 39.2 mg of CuSO_4_·5H_2_O, 24.8 mg of Co­(NO_3_)_2_·6H_2_O, and 17.7 mg of Na_2_B_4_O_7_·10H_2_O.

### MBR System Configuration

The reactor vessel had a 300 mL capacity (2 Parallel bioreactor system, INFORS-HT, Bottmingen, Switzerland), and system conditions including pH, temperature, stirring speed, air supply, and feed rate were monitored and controlled by the Eve software (Eve bioprocess platform software, INFORS-HT, Bottmingen, Switzerland). A membrane module included a hollow-fiber membrane, two pumps, and a time controller. The hollow-fiber membrane, which has a pore size of 0.2 μm and a surface area of 290 cm^2^ (Repligen D04-P20U-05-S, Waltham, MA, USA), was connected to the reactor via a recirculation loop. One pump was connected to the recirculation loop, through which the liquid and cells in the reactor were circulated through the membrane lumen. Another pump was connected to the filtrate port of the membrane, where the soluble effluent was collected. The time controller (Chrontrol XT-4S, Chrontrol, San Diego, CA, USA) regulated these pumps to control the HRT of the system. All MBR experiments were performed at 25 °C and pH 7 with continuous aeration at a flow rate of 1 L/min and a stirrer speed between 250 and 320 rpm.

The experimental design for this study consisted of operating the bioreactor system using one configuration, assessing the performance, and then modifying the bioreactor configuration to improve upon the observed performance. Thus, one experiment with one configuration was sufficient to indicate the need for a configuration change to improve the process performance. In the continuous-flow-through MBR system, a 200 mL working volume was maintained via the membrane module. The timer activated the recirculation pump at discrete time intervals of ∼20 min, and it was operated for about 5 min each time. During these periods, the effluent pump was turned on 2 min after activation of the recirculation pump, and the pump rate was set to produce a volume of effluent equal to the volume fed when the system was off. In the batch and step-fed batch experiments, the membrane module was activated only at the end of the batch cycle, i.e., at a 250 mL cumulative working volume. Both the recirculation and effluent pumps were manually turned on and maintained until approximately 200 mL of soluble effluent was produced.

### Preparation of Alkaline Pretreatment Liquor

Five 500 mL autoclave glass bottles were filled with 40 g of biomass each (5% moisture content) and 160 g of 1 M sodium hydroxide (20% solids loading). The mixture was incubated at 121 °C for 1 h. The solution was cooled to room temperature and then acidified with 36% H_2_SO_4_ to reach pH 2.0 and then incubated again at 121 °C for 1 h. Following acidic incubation and cooling to room temperature, the aromatic compounds were extracted using ethyl acetate (EtOAc). For this extraction, 100 mL of distilled water was added to each bottle to suspend the biomass, and then 200 mL of EtOAc was added to each bottle. The contents of all five bottles were mixed well, transferred to a large Büchner funnel with a 20 μm pore size filter paper, and vacuum filtered. The collected liquid was transferred into a separatory funnel and allowed to phase separate into an aqueous solution #1 and an organic solution #1. A second extraction of the biomass was conducted in the Büchner funnel by adding 1000 mL of EtOAc to the biomass, mixing the suspension, and then filtering through the 20 μm filter paper. Then, the EtOAc-saturated biomass was washed in the Büchner funnel with 1000 mL of distilled water and filtered through the 20 μm filter paper. The filtrate from the second EtOAc extraction and the distilled water washing were transferred into a separatory funnel and partitioned into aqueous solution #2 and organic solution #2. The two organic solutions were then combined and rotovaped to give an amber viscous oil. The oil was resuspended in 100 mL of 0.2 M NH_4_OH. This solution was rotovaped for 5 min to remove any residual EtOAc, then filtered through a 0.2 μm filter, and frozen (−20 °C) until further use. The resulting solution was 75 mL and contained 178.5 mM *p*HBA. This solution was then diluted to give the first eAPL used in the experiments and contained 83 mM *p*HBA as the main aromatic compound.

The eAPL2 preparation deviated from the eAPL protocol after the acidic incubation step. In the eAPL2 procedure, the pH of samples was adjusted to 5.5 using 1 M NaOH. Then, the aromatic compounds were extracted following the eAPL procedure above. Instead of evaporating the 1500–1700 mL of EtOAc to a viscous oil, the combined EtOAc solution was transferred to a separatory funnel and extracted 20 times with 200 mL of distilled water, adding additional EtOAc to the funnel to maintain at least 500 mL of EtOAc in the funnel. After the 20 extractions, the EtOAc fraction maintained its initial amber color, indicating retention of some organic component. The combined 4000 mL of water extract was then rotovaped down to 220 mL, which contained 75.2 mM *p*HBA and 18.8 mM acetate. This solution was diluted to give a final eAPL2 solution, which contained 44.3 mM *p*HBA as the main aromatic compound.

### Analytical Methods

The measurement of the aromatic compounds in the culture media or filtrate was done via a triple quadrupole high-performance liquid chromatography–mass spectrometry (HPLC-MS) (Nexera XR HPLC-8045 MS/MS, Shimadzu, Japan).^12^ The mobile phases consisted of Solvent A (0.1% formic acid in HPLC grade water, v/v) and Solvent B (100% methanol). These phases flowed through the column (Kinetex, 2.6 μm F5, 150 mm, USA) at a binary gradient rate of 0.45 mL/min. All compounds were detected by multiple-reaction monitoring (MRM) and quantified using the strongest MRM transition. Astaxanthin and CoQ_10_ were measured via HPLC (Nexera XR, Shimadzu, Japan).^22^ The mobile phases are as follows: Solvent A—70% acetonitrile/30% water; Solvent B, 70% acetonitrile/30% isopropanol. The binary gradient of mobile phases flowed at 0.45 mL/min through the column (2.6 μm PS C18 150 mm, Kinetex, USA). A photodiode array detector (SPD-M20A, Shimadzu, Japan) was used with an absorbance range between 200 and 600 nm. A biochemistry analyzer (YSI 2900, Xylem, USA) was used for glucose measurement. NH_4_^+^ was measured via an Ammonia Hach Kit (TNT 833, Hach, USA) and a spectrophotometer (Hach DR3900, Hach, USA).

### Astaxanthin and CoQ_10_ Extraction

The concentrated cells were centrifuged at 5000 rpm for 15 min, and the supernatant was discarded. The resulting cell pellets were resuspended in an extraction solvent (7:3 acetone/methanol) by pipetting. The suspended pellets were sonicated for ∼3 min and then centrifuged at 5000 rpm for 15 min. The soluble supernatant was used to measure astaxanthin and CoQ_10_ via HPLC as described above.

## Supplementary Material



## Notes

Methods for the generation of the PDC2SastaW *N. aromaticivorans* mutant; astaxanthin concentration upon extended incubation; astaxanthin accumulation in batch experiments at various glucose concentrations; graphical depiction of the four steps in the SBR-MBR cycle; performance data from step-fed batch experiment with eAPL; and summary of experimental conditions and products during bioreactor experiments (PDF)
