# Supplementary material for: Using Novosphingobium aromaticivorans for Concurrent Production of Intracellular and Extracellular Products from Aromatics Extracted from Poplar Biomass
Source: ACS ES T Eng. 2026 Feb 17;6(3):1106–17. doi: 10.1021/acsestengg.5c00956 (PMC12993862; doi:10.1021/acsestengg.5c00956)
Supplement: Supplementary file 1 [file ee5c00956_si_001.pdf]

Supporting Information

Using *Novosphingobium aromaticivorans* for concurrent production of intracellular and extracellular products from aromatics extracted from poplar biomass

Bumkyu Kim,[a,b,c] Benjamin W. Hall,[a,b,c] Dennis V. Haak,[b,c] Jason Coplien,[b,c] Steven D. Karlen,[b,c] Timothy J. Donohue[b,c,d], and Daniel R. Noguera *[a,b,c]

[a]*Department of Civil and Environmental Engineering, University of Wisconsin-Madison, Madison, WI 53706, USA.*
[b]*Wisconsin Energy Institute, University of Wisconsin-Madison, Madison, WI 53726, USA.*
[c]*Great Lakes Bioenergy Research Center, University of Wisconsin-Madison, WI 53726, USA.*
[d]*Department of Bacteriology, University of Wisconsin-Madison, WI 53706, USA*

Supplementary Methods

Generation of PDC2SastaW *N. aromaticivorans* mutant

Creation of *N. aromaticivorans* PDC2SastaW was achieved by deleting Saro_2861 (*ligM*) in PDCSastaW[1] using homologous recombination. Regions of the *N. aromaticivorans* genomic DNA containing ~1000 bp upstream and downstream of Saro_2861 were amplified via PCR. Plasmid pK18msB-MCS1 (a variant of pK18*mobsacB*[2] in which the multiple cloning site has been removed, and which contains a gene for kanamycin resistance and *sacB* for sucrose sensitivity) was linearized via PCR as previously described.[3] The upstream and downstream flanking regions for each gene were combined with linearized pK18msB-MCS1 using the NEBuilder HiFi Assembly system (New England Biolabs, Ipswich, MA) to produce a plasmid in which the upstream and downstream DNA sequences are adjacent, with no intervening coding region. The plasmid was transformed into NEB 5-alpha competent *Escherichia coli* cells (New England Biolabs). The transformed *E. coli* cells were cultured in LB media + kanamycin, the plasmid was purified using a Qiagen® Plasmid Maxi Kit (Qiagen, Germany), and DNA sequencing was used to confirm the presence of the desired junction between upstream and downstream fragments.

Plasmid containing the upstream and downstream regions of homology near Saro_2861 was transferred into *N. aromaticivorans* by conjugation using *E. coli* S17-1 cells. Transconjugants that had recombined the plasmid into the genome were selected for on plates containing kanamycin. Kanamycin-resistant colonies were streaked onto SMB + 10 mM glucose + 10% sucrose (w/v) to select for double crossovers that result in the plasmid looping out of the genome. Colonies on sucrose-containing plates were patched onto kanamycin and sucrose plates separately to screen for kanamycin sensitivity and sucrose resistance. Such colonies were then screened by colony PCR to check for the desired genetic mutation, which was confirmed by Sanger sequencing.

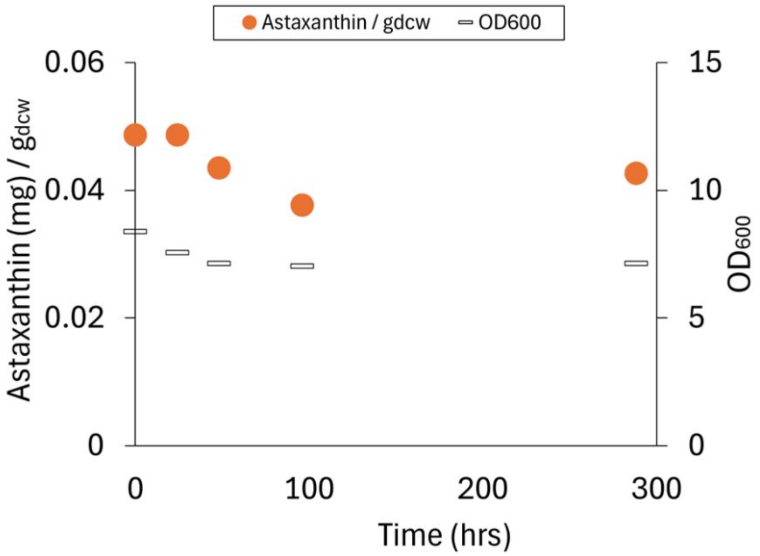

Figure S1. *Batch results for endogenous respiration of astaxanthin. The figure shows the relative amount of astaxanthin and OD_{600}. Data was collected during the first 100 hours, and then, at the end of the ~ 300-hour experiment.*

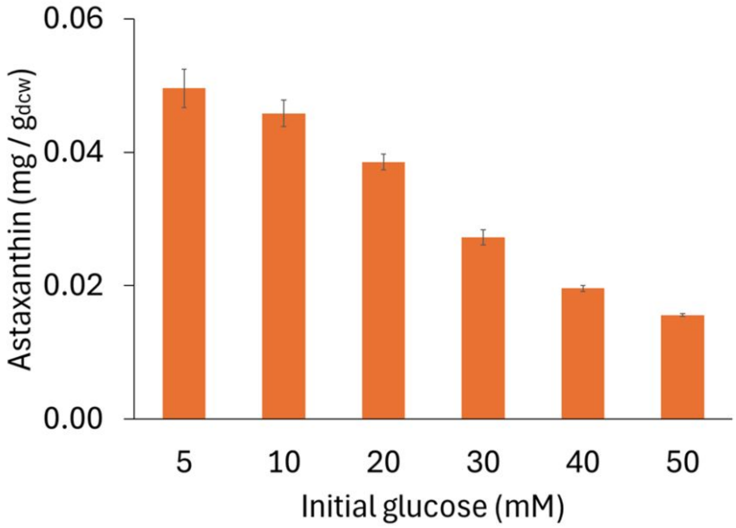

Figure S2. *Batch results for astaxanthin accumulation at various glucose concentrations. Each glucose concentration on the X-axis began with the same initial cell density, creating various glucose to cell ratios at each condition. The Y-axis represents the relative amount of astaxanthin normalized to cell concentration at the end of the batch (i.e., the final sample).*

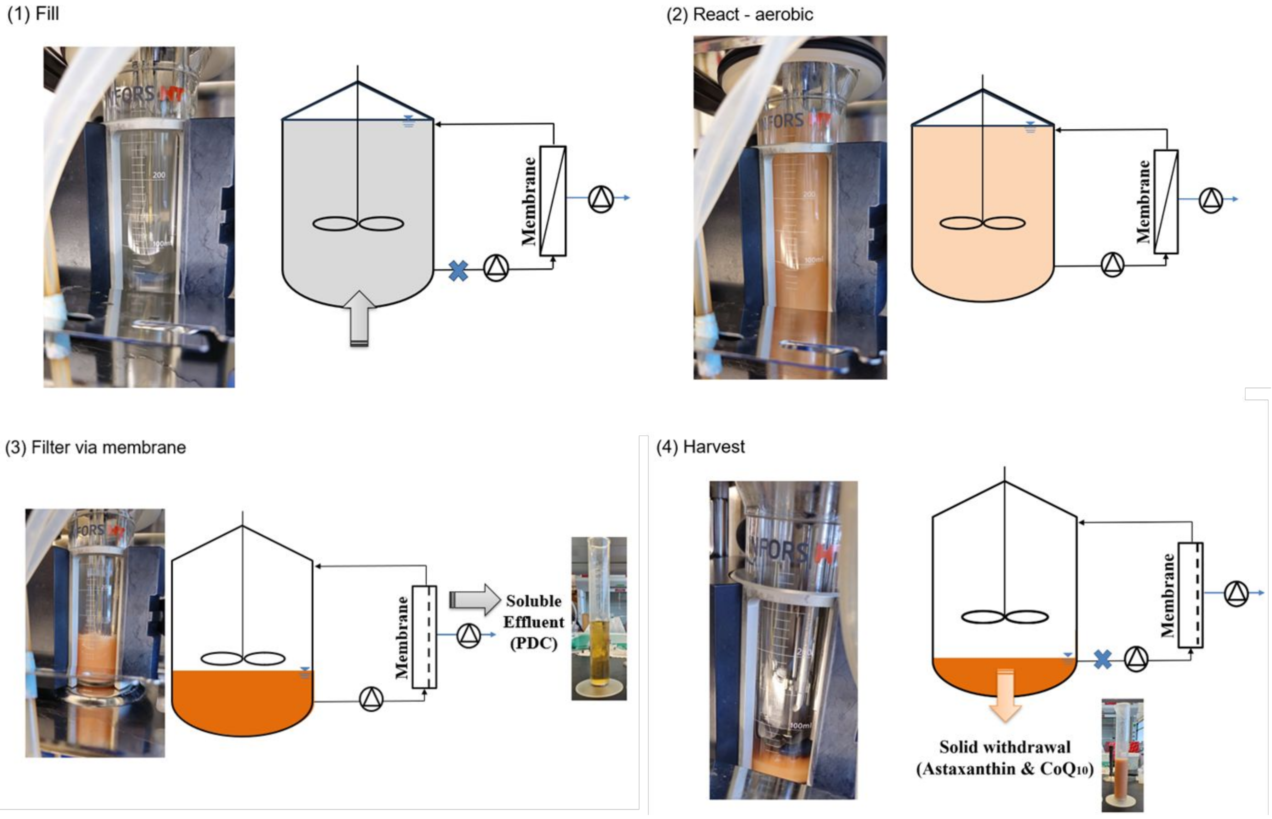

Figure S3. *Graphical depiction of the four steps in the SBR-MBR cycle: Fill, react, filter, and harvest. The fill step can be rapid addition of the substrate or step-feed mode. During the react cycle, the vessel is aerated and the duration of the step depends on the rate of oxygen consumption. The membrane module is only used in the filter step.*

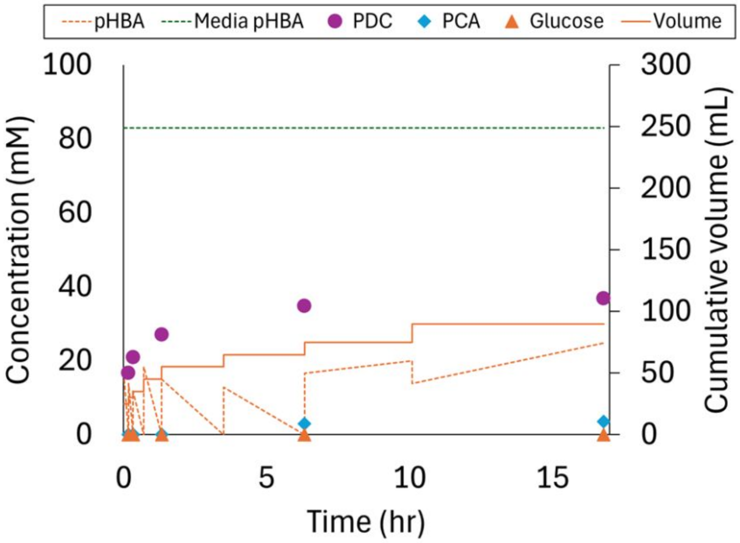

Figure S4. *Performance data for a step-fed batch flow-through MBR fed the media of alkaline pretreated liquid from poplar including pHBA, glucose, and phosphate buffer using NH_4OH for pH control. The fed-batch experiment used eAPL with 83 mM pHBA and 100 mM glucose. The figure shows the concentration of pHBA, media pHBA, PCA, PDC, glucose and cumulative volume throughout the fed-batch period.*

References

1. Hall, B. W.; Kontur, W. S.; Neri, J. C.; Gille, D. M.; Noguera, D. R.; Donohue, T. J., Production of carotenoids from aromatics and pretreated lignocellulosic biomass by *Novosphingobium aromaticivorans*. *Appl. Environ. Microbiol.* **2023,** *89* (12), 1-16.

2. Schäfer, A.; Tauch, A.; Jäger, W.; Kalinowski, J.; Thierbach, G.; Pühler, A., Small mobilizable multi-purpose cloning vectors derived from the *Escherichia coli* plasmids pK18 and pK19: selection of defined deletions in the chromosome of *Corynebacterium glutamicum*. *Gene* **1994,** *145* (1), 69-73.

3. Kontur, W. S.; Bingman, C. A.; Olmsted, C. N.; Wassarman, D. R.; Ulbrich, A.; Gall, D. L.; Smith, R. W.; Yusko, L. M.; Fox, B. G.; Noguera, D. R.; Coon, J. J.; Donohue, T. J., *Novosphingobium aromaticivorans* uses a Nu-class glutathione *S*-transferase as a glutathione lyase in breaking the β-aryl ether bond of lignin. *J. Biol. Chem.* **2018,** *293* (14), 4955-4968.

Table S1. Summary of experimental conditions and products during bioreactor experiments

Experiment	Figure	Operation Mode	Flow Rate (mL/hr)	HRT (hr)	Influent			End of cycle						
					Glucose (mM)	*p*HBA (mM)	NH_4^+ (mM)	Astaxanthin (mg/g_{dcw})	CoQ10 (mg/g_{dcw})	PDC (mM)	PCA (mM)	pHBA (mM)	Cell density (OD_{600})
1_1	1	Continuous flow	8.3	24	50	0	0	0.036	0.23	NA	NA	NA	9
1_2	1	Continuous flow	16.7	12	50	0	0	0.006	0.03	NA	NA	NA	41
1_3	1	Continuous flow	33.3	6	50	0	0	0.006	0.05	NA	NA	NA	43
2_1	2	SBR-MBR	NA	NA	50	0	0	0.043	0.43	NA	NA	NA	11
2_2	2	SBR-MBR	NA	NA	50	0	0	0.035	0.43	NA	NA	NA	12
2_3	2	SBR-MBR	NA	NA	50	0	0	0.035	0.38	NA	NA	NA	11
2_4	2	SBR-MBR	NA	NA	50	0	0	0.043	0.45	NA	NA	NA	11
2_5	2	SBR-MBR	NA	NA	50	0	0	0.048	0.49	NA	NA	NA	12
3_1	3	SBR-MBR step-feed	NA	NA	50	0	0	0.043	0.41	0	0	0	11
3_2	3	SBR-MBR step-feed	NA	NA	50	2.0	3.1	0.042	0.45	1.8	0	0	12
3_3	3	SBR-MBR step-feed	NA	NA	50	10.0	11.0	0.045	0.43	10.5	0	0	13
3_4	3	SBR-MBR step-feed	NA	NA	50	15.0	15.4	0.042	0.42	14.4	0	0	11
3_5	3	SBR-MBR step-feed	NA	NA	50	23.0	22.1	0.033	0.35	23.0	0	0	14

Experiment	Figure	Operation Mode	Flow Rate (mL/hr)	HRT (hr)	Influent			End of cycle					
					Glucose (mM)	*p*HBA (mM)	NH_4^+ (mM)	Astaxanthin (mg/g_{dcw})	CoQ10 (mg/g_{dcw})	PDC (mM)	PCA (mM)	pHBA (mM)	Cell density (OD_{600})
3_6	3	SBR-MBR step-feed	NA	NA	50	25	24.1	0.027	0.41	24.2	0	0	12
3_7	3	SBR-MBR step-feed	NA	NA	50	30	29.1	0.025	0.46	19.2	10.7	0	10
3_8	3	SBR-MBR step-feed	NA	NA	50	35	34.5	0.021	0.41	12.6	20.0	4.0	6
4_1	4	SBR-MBR step-feed	NA	NA	45	30	32.1	0.029	0.45	26.1	0	0	17
4_2	4	SBR-MBR step-feed	NA	NA	65	55	57.1	0.026	0.44	53.1	0	0	19
4_3	4	SBR-MBR step-feed	NA	NA	90	80	84.3	0.023	0.43	77.6	0	0	19
4_4	4	SBR-MBR step-feed	NA	NA	110	100	119.9	0.017	0.40	88.6	10.1	0	30
5_1	S4	SBR-MBR step-feed	NA	NA	100	83 (in eAPL)	343	NA	NA	36.8	3.52	22.5	19.6
6	5	SBR-MBR step-feed	NA	NA	50	44 (in eAPL2)	50	0.03	0.44	42.4	0	0	16
